# Characterization of Mixed-Species Biofilms Formed by Four Gut Microbiota

**DOI:** 10.3390/microorganisms10122332

**Published:** 2022-11-25

**Authors:** Tao Xu, Yue Xiao, Hongchao Wang, Jinlin Zhu, Yuankun Lee, Jianxin Zhao, Wenwei Lu, Hao Zhang

**Affiliations:** 1 State Key Laboratory of Food Science and Technology, Jiangnan University, Wuxi 214122, China; 2 School of Food Science and Technology, Jiangnan University, Wuxi 214122, China; 3 Department of Microbiology & Immunology, Yong Loo Lin School of Medicine, National University of Singapore, Singapore 117545, Singapore; 4 International Joint Research Laboratory for Pharmabiotics & Antibiotic Resistance, Jiangnan University, Wuxi 214122, China; 5 (Yangzhou) Institute of Food Biotechnology, Jiangnan University, Yangzhou 225004, China; 6 National Engineering Research Center for Functional Food, Jiangnan University, Wuxi 214122, China

**Keywords:** gut microbiota, mixed-species biofilms, *Bacteroides*, *Bifidobacterium*, *Enterococcus*, *Lactobacillus*, synergistic interaction

## Abstract

In natural settings, approximately 40–80% of bacteria exist as biofilms, most of which are mixed-species biofilms. Previous studies have typically focused on single- or dual-species biofilms. To expand the field of study on gut biofilms, we found a group of gut microbiota that can form biofilms well in vitro: *Bifidobacterium longum* subsp. *infantis*, *Enterococcus faecalis*, *Bacteroides ovatus*, and *Lactobacillus gasseri*. The increase in biomass and bio-volume of the mixed-species biofilm was confirmed via crystal violet staining, field emission scanning electron microscopy, and confocal laser scanning microscopy, revealing a strong synergistic relationship in these communities, with *B. longum* being the key biofilm-contributing species. This interaction may be related to changes in the cell number, biofilm-related genes, and metabolic activities. After quantifying the cell number using quantitative polymerase chain reaction, *B. longum* and *L. gasseri* were found to be the dominant flora in the mixed-species biofilm. In addition, this study analyzed biological properties of mixed-species biofilms, such as antibiotic resistance, cell metabolic activity, and concentration of water-insoluble polysaccharides. Compared with single-species biofilms, mixed-species biofilms had higher metabolic activity, more extracellular matrix, and greater antibiotic resistance. From these results, we can see that the formation of biofilms is a self-protection mechanism of gut microbiota, and the formation of mixed-species biofilms can greatly improve the survival rate of different strains. Finally, this study is a preliminary exploration of the biological characteristics of gut biofilms, and the molecular mechanisms underlying the formation of biofilms warrant further research.

## 1. Introduction

Bacteria generally exist as biofilms [1]. Biofilms were first defined by Costerton [2]. Simply, “biofilm” refers to an organized community of bacteria that is attached to the surface of some biotic or abiotic carriers. In biofilms, these bacteria are generally coated with extracellular polymers (EPSs), such as polysaccharides, proteins, and nucleic acids [3]. Biofilm is a very complex microbial ecosystem that represents a self-protective mode of various strains and can be seen as a miniature metabolic factory [4]. Compared to single cells, the growth rate, gene expression, living habits, and structural appearance of each member in mixed-species biofilms are significantly different, which are mainly reflected in the improvement in biofilm metabolic capacity, environmental stress tolerance, and community-level signaling [5,6,7].

Mixed-species biofilms exist in many ecological niches, such as the ocean, human oral cavity and gut, and soil. For example, biofilms formed by *Listeria monocytogenes*, *Klebsiella*, *Escherichia coli*, *Comamonas* sp., and *Acinetobacter* sp. were found in dairy environments [8]; *Streptococcus mutans*, *Streptococcus sanguis*, and *Streptococcus gordonii* were found in dental plaque biofilms [9]; and six species biofilms, which were composed of *Cupriavidus metallidurans*, *Sphingomonas paucimobilis*, *Chryseobacterium gleum*, *Ralstonia pickettii*, *Methylorubrum populi*, and *Ralstonia insidiosa*, were found in drinking-water recycling systems [10].

There is extensive evidence that biofilms exist in the human gut; these biofilms generally attach to food particles in the colon or colonize in the mucus layer in the gut lumen [11,12,13]. However, mixed-species biofilms formed in the gut can cause severe disease under certain circumstances [14,15]. For commensal bacteria, their biofilm formation can not only effectively maintain the stability and resilience of the intestinal microenvironment but also have a significant effect on resisting the invasion of intestinal pathogens. Therefore, studying the mechanism of biofilm formation in the gut is critical for exploring the interactions among the gut microbiota and preventing serious infections.

To date, studies on gut biofilms have mainly focused on single or dual species, but little is known about mixed biofilms. Given the current state of research on gut biofilms, the aim of this study was to identify a group of key biofilm-formation strains through an in vitro static culture system based on the potential cross-feeding relationship of the gut microbiota. Then, the changes in biomass, structure, metabolic activity, EPS formation, and differences in antibiotic resistance of the biofilms, which were composed of the identified community, will be explored.

## 2. Materials and Methods

### 2.1. Strains and Culture Conditions

Four species were used in this study to establish biofilms: *Bifidobacterium longum* subsp. *Infantis* FJND16M4, *Bifidobacterium longum* subsp. *Infantis* FBJCP1M11, *Enterococcus faecalis* E1, *Enterococcus faecalis* JNGMMB7, *Bacteroides ovatus* FTJS5K9, *Bacteroides ovatus* FNXYCHL6K1, *Lactobacillus gasseri* FHNFQ11L7, and *Lactobacillus gasseri* FHNFQ14L5. All were isolated from stool samples and obtained from the Research Center of Food Biotechnology, JiangNan University, WuXi, China. All strains were cultured in YCFA medium with some modifications [16,17]. Before the establishment of biofilms, strains were propagated for three generations to obtain strong cell vitality in an anaerobic incubator statically at 37 °C in a CO_2_, H_2_, and N_2_ environment. Details of the strains and media are presented in Tables S1 and S2, respectively.

### 2.2. Culture of Biofilms

These strains were incubated anaerobically overnight until exponential phase and the culture was diluted with fresh mYCFA medium to an optical density of 0.05 (OD_600nm_), so that they had approximately the same starting inoculum cells (1 × 10^3^ CFU/mL) [18]. To explore the synergistic effect on the biomass of mono- and mixed-species biofilms, all possible combinations of these four strains were developed. In short, for mono-biofilms, 200 μL of the diluted culture was added to 96-well microplates (Sorfa Life Science Research, Co., Ltd., Huzhou, China). Each group was independently analyzed three times on different occasions, with three biological replicates per experiment (3 × 3). Each species of mixed-biofilm was mixed at the same volume ratio, and the microplate was incubated in a 37 °C anaerobic incubator at a static state for 24 h.

### 2.3. Effect of Cell-Free Supernatant on the Formation of Biofilms

The cell-free supernatant of each strain was obtained from overnight cultures by filtration through 0.22 μm filters (Sorfa Life Science Research, Co., Ltd., Huzhou, China) [19]. The diluted culture of viable cells and the cell-free supernatant were added to 96-well microplates at the same volume ratio. The total inoculum volume of mixture was 200 μL. Then, the biofilms were incubated under anaerobic conditions at 37 °C for 24 h.

### 2.4. Quantification of Biofilms through Crystal Violet Staining (CV)

The CV staining method used in this study was previously reported [20]. In brief, after removing the suspension in wells, mature biofilms in 96-well microplates were carefully washed twice with 0.1 M PBS to remove planktonic cells and fixed with methanol solution (Sinopharm Chemical Reagent, Co., Ltd., Shanghai, China) for 15 min. Then, biofilms were stained with 0.1% (*w*/*v*) CV solution (Sinopharm Chemical Reagent, Co., Ltd., Shanghai, China) for 30 min and washed with 0.1 M PBS to remove the CV stain solution. Finally, the microplates were dried and 200 μL of 33% acetic acid was added and incubated at 20 °C for 30 min. For each well, 200 μL of the solution was transferred into new 96-well microplates. The biomass of biofilms was quantified using optical density measured by Multiskan GO (Thermo Fisher Scientific, Waltham, MA, USA) at 590 nm.

### 2.5. Quantification of the Cell in Suspension and Biofilms through qPCR

The cell count of each strain in biofilms and suspensions was quantified using qPCR [21]. Briefly, all biofilms were developed in 24-well microplates (Sorfa Life Science Research, Co., Ltd., Huzhou, China) and the starting inoculum volume of mixture in each well was 1 mL. After the incubation of biofilms, 1 mL of the suspension was transferred into 1.5 mL centrifuge tubes and the biofilms were scraped through pipette tips repeatedly to ensure that the biofilms were resuspended in 1 mL PBS completely. Three biological replicates were used at each time point. Subsequently, the cells were collected by centrifugation at 9000 rpm for 4 min and stored at −80 °C.

Total DNA was extracted with the TIANamp Bacteria DNA Kit (Tiangen Biotech Beijing, Co., Ltd., Beijing, China) [18]. During the process of creating the standard curve, TES solution was used to sequentially dilute the DNA extracted from each bacterial culture according to a 10-fold gradient (10^−1^~10^−8^). The final PCR reaction volume was 25 μL, including 2 μL of the DNA template, 8.5 μL of ddH_2_O, 12.5 μL of SYBR Green Premix Ex Taq II (Bio-Rad, Co., Ltd., Hercules, CA, USA), and 1 μL of each primer (Sunny Biotechnology, Co., Ltd., Shanghai, China). The PCR program was as follows: 95 °C for 30 s, followed by 40 cycles of 95 °C for 5 s and 60 °C for 30 s. The melt curve was determined as follows: 65 °C, 5 s; 95 °C; 0.5 °C. Details of each strain-specific primer are listed in Table S3.

### 2.6. Analysis of Biofilm Metabolic Activity

The metabolic activity of cells in biofilms was determined using the XTT reduction method as previously reported [22]. Briefly, the 1 mg/mL XTT solution (Macklin Biochemical Technology, Co., Ltd., Shanghai, China) was prepared with PBS and the 0.4 mM menadione solution (Macklin Biochemical Technology, Co., Ltd., Shanghai, China) was prepared with acetone (Sinopharm Chemical Reagent, Co., Ltd., Shanghai, China).

The biofilms were cultured in 24-well microplates as mentioned above. The total starting inoculum volume was 1 mL. After incubation, the biofilms were washed with 0.1 M PBS twice to remove suspended cells. Next, 10 μL of menadione solution, 200 μL of XTT solution, and 790 μL of phosphate buffer were added to each well of a 24-well microplate. The microplates were incubated at 37 °C, while being protected from light, for 3 h. Finally, we transferred 200 μL of mixed solution in each well to a new 96-well microplate. The changes in metabolic activity of each sample were measured through the optical density using Multiskan GO at 492 nm.

### 2.7. Quantitative Analysis of Water-Insoluble Polysaccharides

The water-insoluble polysaccharide assay was performed through the anthrone-sulfuric acid method as previously reported [23,24]. Briefly, the biofilms were cultured in 24-well microplates and the total starting inoculum volume was 1 mL. After washing with 0.1 M PBS, the biofilms were scraped completely through pipette tips and resuspended in 1.5 mL centrifuge tubes containing 1 mL of 0.1 M PBS. The tubes were centrifuged at 6000× *g* for 10 min to remove the supernatant, and this step needed to be carried out three times. Then, 1 mL of 1 M NaOH was added to each centrifuge tube and stirred fully for two hours to ensure that all water-insoluble polysaccharides were extracted. The carbohydrate solution and anthrone-sulfuric acid (Sinopharm Chemical Reagent, Co., Ltd., Shanghai, China) were mixed at a ratio of 1:3 (*v*/*v*). After that, the mixture was heated at 95 °C for 5 min and cooled to room temperature. The exopolysaccharide concentration of biofilms was determined using a pre-measured standard curve and optical density, which was measured at 625 nm using Multiskan GO.

### 2.8. Assay of Ability of Biofilm Antibiotic Resistance

Antibacterial drug susceptibility can be analyzed through minimum biofilm inhibitory concentration (MBIC) experiments [25,26]. After the incubation of biofilms in 96-well microplates, we removed the suspension and washed the biofilms twice. Then, we added 100 μL of sterile antibiotic solution to the microplates and incubated at 37 °C for 24 h. Sterile water was added to the positive group and fresh medium was set as the negative group. After antibiotic intervention, we removed the suspension and added 200 μL of fresh mYCFA medium into each well, followed by incubation at 37 °C for 24 h. Finally, the optical density was measured at 600 nm using Multiskan GO. The concentration at which no bacterial growth was detected was considered as the MBIC.

### 2.9. Observation of Biofilms through FESEM

The biofilms were incubated on coverslips (24 × 24 mm) (Citotest Labware Manufacturing Co., Ltd., Haimen, China) in 6-well microplates (Sorfa Life Science Research, Co., Ltd., Huzhou, China) at 37 °C for 24 h and the starting inoculum volume of biofilms was 5 mL. Subsequently, we removed the planktonic cells through washing the coverslips twice and fixed the biofilms overnight at 4 °C with 2.5% glutaraldehyde solution (SenBeiJia Biological Technology, Co., Ltd., Nanjing, China). Then, the biofilms were dehydrated using 15%, 30%, 50%, 70%, 80%, 90%, and 100% ethanol (10 min intervals). Finally, each sample was coated with gold and analyzed using the software provided by the FESEM (Regulus8100, HITACHI, Tokyo, Japan) [27].

### 2.10. Observation of Biofilms through CLSM

The biofilms were incubated on coverslips (24 × 24 mm) in 6-well microplates at 37 °C for 24 h and the starting inoculum volume of biofilms was 5 mL. After incubation, we removed the planktonic cells and stained the biofilms with SYBR Green I (Sangon Biotech Co., Ltd., Shanghai, China). Then, we washed all samples twice with PBS to remove fluorescent dyes and air-dried slightly. The dyeing process must be protected from light. Images were obtained using a confocal laser scanning microscope (LSM710, Carl Zeiss AG, Oberkochen, Germany). A 20× objective was used to capture the fluorescence excited at 488 nm and emitted at 500–550 nm. The software to capture CLSM images was “ZEN black edition”. Structural parameters related to the biofilm structure were extracted and analyzed from the three-dimensional images of CLSM by the Comstat2.1 software [28,29].

### 2.11. Statistical Analysis

Data analysis was performed using Microsoft Excel 2019 and GraphPad Prism v.8 software (GraphPad Software Inc., San Diego, CA, USA). Differences with *p*-values < 0.05 were considered statistically significant.

## 3. Results

### 3.1. Formation Ability of Mono- and Mixed-Species Biofilms

**Figure 1:**
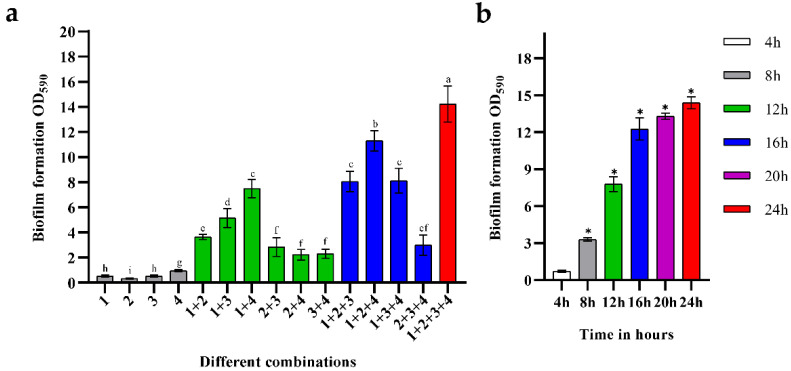
(**a**) Biofilm formation of the four strains. The numbers marked in the histogram represent different strains: 1—*B. longum subsp. Infantis* FJND16M4, 2—*B. ovatus* FTJS5K9, 3—*L. gasseri* FHNFQ11L7, and 4—*E. faecalis* E1. Lowercase letters above bars represent significant differences in biofilm mass between combinations (*p* < 0.05) after one-way ANOVA. (**b**) The biomass of mixed biofilms composed of *B. longum* FJND16M4, *B. ovatus* FTJS5K9, *L. gasseri* FHNFQ11L7, and *E. faecalis* E1 at different time points (4 h/8 h/12 h/16 h/20 h/24 h). The asterisks above the bar represent significant difference in biofilm mass between this point and the previous time point (*p* < 0.05) after one-way ANOVA. Error bars in the figure represent ±SEM of biological replicates.

Figure 1a shows the biomass of biofilms composed of *B. longum* FJND16M4, *L. gasseri* FHNFQ11L7, *E. faecalis* E1, and *B. ovatus* FTJS5K9. Images of the crystal violet staining of different combinations are shown in Figure S1. The difference in the biomass of the biofilms demonstrated strong synergistic interactions between different strains. Among the mono-biofilms, *E. faecalis* had biofilm-formation ability, while *B. longum*, *L. gasseri*, and *B. ovatus* could not form abundant biofilms when cultured alone. When these four strains were co-cultured, the biomass of the mixed-species biofilms increased significantly, approximately 5.9-times of the sum of the mono-biofilms. Among the combinations of two strains, the synergistic effect on biofilms composed of *B. longum* + *E. faecalis* was the most significant, and the biomass was approximately 4.8-times higher than that of mono-biofilms. The combination of *B. longum* + *B. ovatus* + *E. faecalis* had the highest biomass in the biofilms of three strains, which was about 6.1-times higher than the total biomass of these single strains. There were also some combinations where the increase in biofilm mass was not significant, such as *L. gasseri* + *B. ovatus* + *E. faecalis*, the biomass of which was only approximately 1.6-times higher than that of mono-biofilms.

Figure 1b shows the changes in the biofilm mass of the combinations of these four strains. For their mixed-species biofilm, the growth rate was significantly quick from 0 to 16 h, indicating that the cross-feeding interaction between strains strongly contributed to their growth in this community. After 16 h, the growth rate slowed, showing that the mixed-species biofilm gradually entered a mature stage.

### 3.2. Interspecies Universality in the Formation of Mixed-Species Biofilms

**Figure 2:**
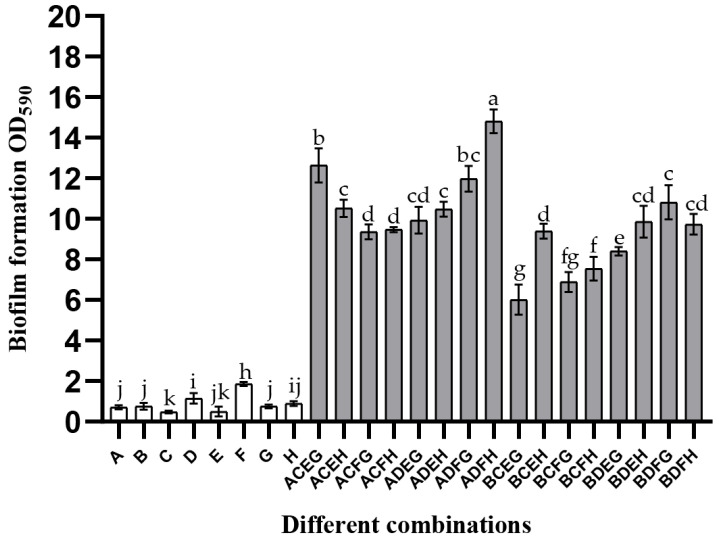
Strain specificity of the mixed-species biofilm. The uppercase letters A–H represent different strains of these four species: A—*B. longum* FJND16M4, B—*B. longum* FBJCP1M11, C—*E. faecalis* E1, D—*E. faecalis* JNGMMB7, E—*B. ovatus* FTJS5K9, F—*B. ovatus* FNXYCHL6K1, G—*L. gasseri* FHNFQ11L7, and H—*L. gasseri* FHNFQ14L5. Different strains of these four species were co-cultured in various combinations to verify the interspecies universality of the mixed-species biofilm formation ability. Error bars in the figure represent ±SEM of biological replicates. Lowercase letters above bars represent significant differences in biofilm mass between combinations (*p* < 0.05) after one-way ANOVA.

This study co-cultured different strains of these four bacteria to explore whether the synergistic interaction was interspecies universal in mixed-species biofilms. Details of the strains used in this experiment are listed in Table S1. It can be seen from the quantification results of CV staining (Figure 2) that the biomass of all mono-biofilms was less than two (OD_590nm_). Although the biomass of a combination of *B. longum* B + *E. faecalis* C + *B. ovatus* E + *L. gasseri* G was approximately six, its biofilm mass increased by 2.23-times compared to the sum of mono-species biofilms. In addition, most of the mixed-species biofilm mass was higher than 10, indicating that the promotion of these species in the process of biofilm formation was species universal.

### 3.3. Effects of Cell-Free Supernatant on Mixed-Species Biofilms

**Figure 3:**
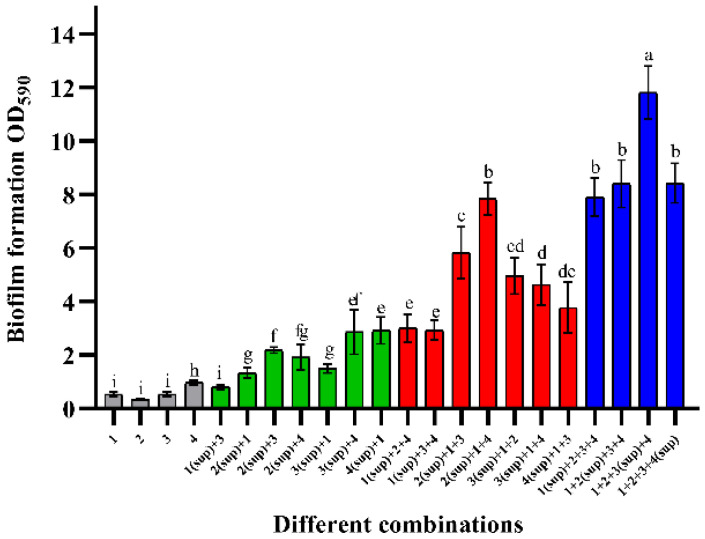
Effect of cell-free supernatant on the formation of mixed-species biofilms. Numbers marked with “sup” represent the cell-free supernatant. The numbers 1–4 represent different species: 1—*B. longum* FJND16M4, 2—*B. ovatus* FTJS5K9, 3—*L. gasseri* FHNFQ11L7, and 4—*E. faecalis* E1. For each sample, diluted cultures and cell-free supernatants of these strains were inoculated into microplates at an equal ratio (*v*/*v*). Error bars in the figure represent ±SEM of biological replicates. Lowercase letters above bars represent significant differences in biofilm mass between combinations (*p* < 0.05) after one-way ANOVA.

Figure 3 shows the changes in biofilm mass when cell-free supernatants were co-cultured with viable cells. By comparing Figure 3 with Figure 1a, it can be seen that the cell-free supernatant did not promote an increase in biofilm mass as significant as that of viable cells. When the supernatant was co-cultured with one viable cell, the biofilm composed of *E. faecalis* (sup) + *B. longum* had the highest biomass, which was approximately 1.95-times that of the sum of their mono-biofilms. Similarly, *B. longum* + *B. ovatus* (sup) + *E. faecalis* had the strongest biofilm formation ability in the combinations of two viable cells. Their biofilm mass was about 4.26-times that of mono-biofilms. Among the three viable strain groups, the combination, which was co-cultured with the *L. gasseri* supernatant, had the highest biomass, approximately 12 (OD_590nm_). The total biofilm mass of this combination was almost 4.97-times that of the sum of their mono-biofilms.

### 3.4. Quantification of Single Bacterial Cell Numbers in Mixed-Species Biofilms

**Figure 4:**
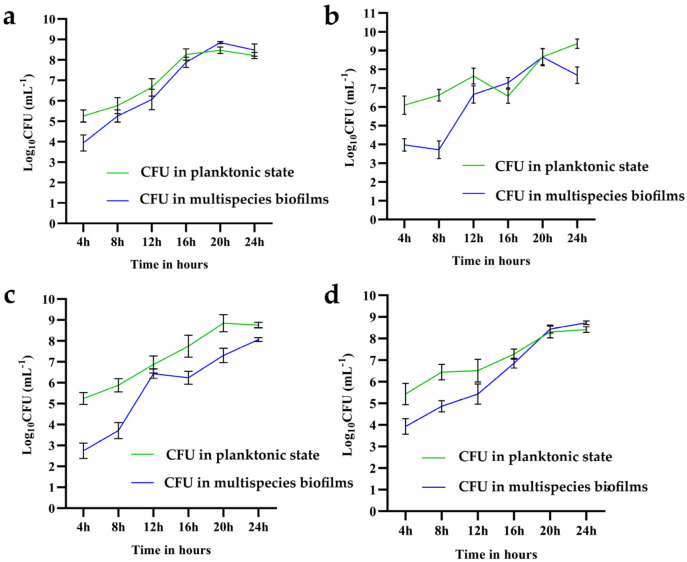
Cell count of the four species in suspension and mixed-species biofilms. (**a**) *B. longum* FJND16M4, (**b**) *E. faecalis* E1, (**c**) *B. ovatus* FTJS5K9, and (**d**) *L. gasseri* FHNFQ11L7. The green line represents the cell number of each species in suspension and the blue line represents the number of each species in mixed-species biofilms. The number of cells was measured every four hours and error bars in the line chart represent the mean value of biological replicates ± SD.

It can be seen from the results in Figure 4a–d that the cell number of *B. longum* increased from 0 to 16 h continuously, after which the growth rate stabilized. After 20 h, the cell number of *B. longum* in biofilms exceeded that in the suspension. However, *B. ovatus* primarily exists as planktonic cells. It went through a period of growth plateau in the mixed-species biofilm during 12–16 h and the growth rate tended to be rapid after 20 h. The numbers of *B. longum* and *B. ovatus* cells in the mixed-species biofilm at 24 h were 8.48 log_10_ CFU/mL and 8.06 log_10_ CFU/mL, respectively. For *L. gasseri*, its cell number in biofilm exceeded that in suspension at approximately 20 h and the final result at 24 h was 8.41 log_10_ CFU/mL (sup) and 8.72 log_10_ CFU/mL, respectively. Between 20 and 24 h, the mixed-species biofilm appeared to be unfavorable for the growth of *E. faecalis*; therefore, it detached from the biofilm into the suspension. The cell number of *E. faecalis* in suspension and biofilm at 24 h was 9.36 log_10_ CFU/mL and 7.69 log_10_ CFU/mL, respectively. In summary, *B. longum* and *L. gasseri* were dominant in the mixed-species biofilm at 24 h, followed by *B. ovatus* and *E. faecalis*.

### 3.5. Analysis of Metabolic Activity and Extracellular Water-Insoluble Polysaccharide of Biofilms

**Figure 5:**
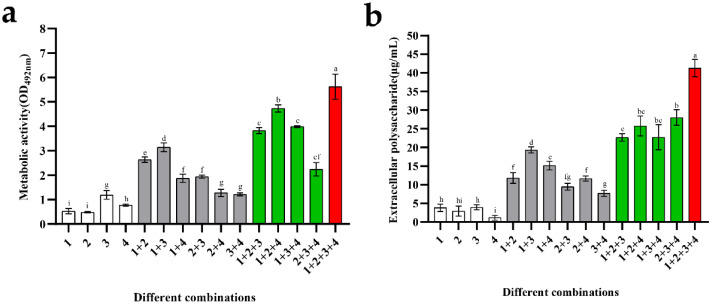
Metabolic activity and exopolysaccharide concentration of mono- and mixed-species biofilms. Numbers in these graphs from 1 to 4 represent the following species: 1—*B. longum* FJND16M4, 2—*L. gasseri* FHNFQ11L7, 3—*E. faecalis* E1, and 4—*B. ovatus* FTJS5K9. Error bars in these graphs represent mean ± SEM of biological replicates. Lowercase letters above the bars represent statistically significant differences between these combinations (*p* < 0.05) after one-way ANOVA. (**a**) Metabolic activity of biofilms. (**b**) The concentration of exopolysaccharide of biofilms.

The cell metabolic activity and concentration of water-insoluble polysaccharides in mono-biofilms were compared with those of mixed-species biofilms. As shown in Figure 5a, *B. longum*, *L. gasseri*, and *B. ovatus* had low metabolic activities, which was mainly related to their poor biofilm formation ability. *E. faecalis* had biofilm-forming ability; therefore, its cell metabolic activity was higher than that of the other strains, with an average value of 1.2 (OD_492nm_). Among the biofilms composed of the two strains, the cell metabolic activity of the combination of *B. longum* + *E. faecalis* was particularly high, with an average value of 3.14. For the combinations composed of the three strains, the presence of *B. longum* promoted an increase in the metabolic activity of biofilms; this was similar to the result of biofilm mass. *B. longum* + *L. gasseri* + *B. ovatus* showed the strongest metabolic activity, with an average value of 4.73. Finally, the biofilms composed of these four strains had the highest metabolic activity (approximately 5.6), which was mainly attributed to the increase in biomass.

Water-insoluble exopolysaccharides are components of the biofilm matrix. From the quantitative results of biofilms (Figure 5b), it can be seen that the exopolysaccharide concentration of *B. ovatus* was particularly low, with an average value of approximately 1.18 μg/mL. Moreover, the content of *B. longum* and *L. gasseri* biofilms was slightly higher than *B. ovatus*: approximately 3.84 μg/mL and 2.92 μg/mL, respectively. The biofilm-formation ability of *E. faecalis* was stronger than that of the other species, while its exopolysaccharide content was still very low (approximately 3.95 μg/mL). For the combinations composed of the two species, their exopolysaccharide content was similar, approximately 10–15 μg/mL. *B. longum* + *E. faecalis* had the most exopolysaccharide (approximately 19.33 μg/mL), which was consistent with the result of their metabolic activity. Among the combinations composed of three species, the quantitative results of these groups were roughly the same, ranging from 22 to 28 μg/mL. When *B. longum*, *L. gasseri*, *E. faecalis*, and *B. ovatus* were co-cultured, mixed-species biofilms had the most exopolysaccharide, with a concentration of about 41.26 μg/mL, indicating powerful metabolic ability and protection of these species.

### 3.6. Changes in Biomass of Mixed-Species Biofilms in Response to Environmental Stress

Gut flora may encounter various environmental stressors in the gastrointestinal tract. For example, acid stress, osmotic pressure, and nutrient stress are known to induce biofilm formation. Therefore, this study explored the effects of different stressors on the biomass of mixed-species biofilms using established biofilm quantification methods.

**Figure 6:**
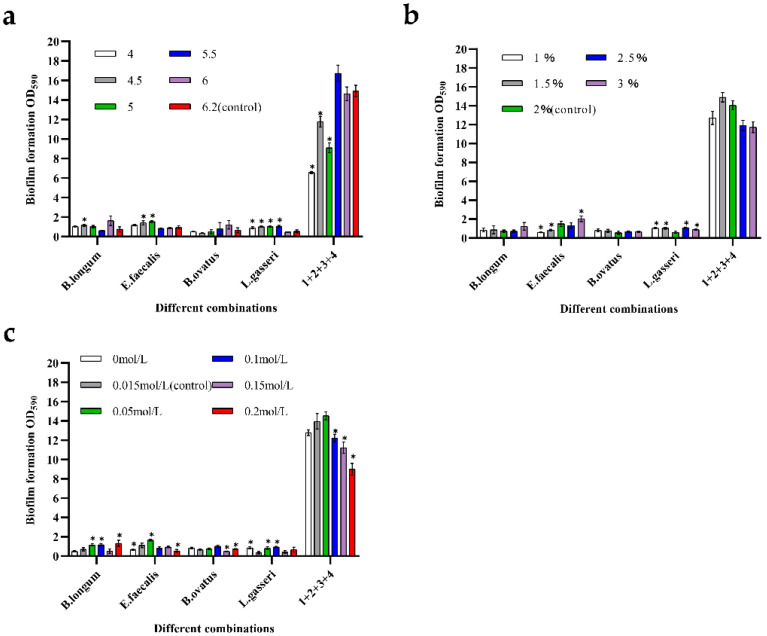
Formation ability of mono- and mixed-species biofilms when exposed to environmental stress. Numbers in these graphs from 1 to 4 represent the following strains: 1—*B. longum* FJND16M4, 2—*E. faecalis* E1, 3—*B. ovatus* FTJS5K9, and 4—*L. gasseri* FHNFQ11L7. Error bars in these graphs represent mean ± SEM of biological replicates and asterisks above the bars mean significant statistical difference between treated groups and control groups (*p* < 0.05) after one-way ANOVA. (**a**) Formation ability of biofilms in response to acid (pH = 4, 4.5, 5, 5.5, 6, and 6.2). (**b**) Formation ability of biofilms in response to nutrient concentration (glucose concentration = 1%, 1.5%, 2%, 2.5%, and 3%). (**c**) Formation ability of biofilms in response to osmotic pressure (sodium chloride concentration = 0 mol/L, 0.015 mol/L, 0.05 mol/L, 0.1 mol/L, 0.15 mol/L, and 0.2 mol/L).

Figure 6a shows the changes in biofilm mass in response to pH. It can be seen that the biofilm mass of *B. longum* and *B. ovatus* increased under weak acid stress conditions (pH = 6): approximately 2.17- and 1.85-times higher than that of the control groups, respectively. For *E. faecalis* and *L. gasseri*, harsh environmental stress can promote an increase in the biofilm mass. At pH 5, the biomass of *E. faecalis* was the highest: about 1.61-times higher than that of the control group. *L. gasseri* had the highest biofilm mass at pH 5.5, which was about 1.95-times higher than that in the control group. For the mixed-species biofilms, the total biomass sharply decreased under intense-stress conditions. At pH 4, the biomass of the mixed-species biofilms was only half that of the control group. Similarly, when the pH was 4.5 and 5, the biofilm mass was also very low, which may be related to the fact that severe acid stress was not conducive to the growth of cells. However, when the pH was 5.5, the production of mixed-species biofilm was the highest, indicating that the weak acidity stress improved the biofilm formation ability, which was about 1.1-times higher than that of the control group. In general, the synergistic effect among the four species would be weakened at a severely low pH, and the production of mixed-species biofilms would be more stable in weakly acidic environments.

Figure 6b shows the changes in biofilms in response to nutrient stress. For mono-biofilms, there was almost no change in the *B. ovatus* biofilm when cultured with different glucose concentrations, indicating that *B. ovatus* was not sensitive to glucose concentration. The biomass of *B. longum* increased only when the glucose concentration was 3%. However, the promotion effect did not appear to be significant in low-nutrient environments. For *L. gasseri*, biomass increased in both nutrient-deficient and nutrient-sufficient environments, demonstrating strong sensitivity to changes in the concentration of the carbon source. Mixed-species biofilms exhibited high growth stability in the presence of nutrient deficiencies. When the glucose concentrations were 2.5% and 3%, the biomasses of the mixed-species biofilm were relatively low. This result may be due to the overgrowth of bacteria and the excessive accumulation of harmful metabolites.

Figure 6c shows the changes in biofilms in response to osmotic pressure. For *B. ovatus*, changes in osmotic pressure did not significantly increase the biomass. The biofilm mass of *L. gasseri* increased when the concentration of sodium chloride was 0 mol/L, 0.05 mol/L, and 0.1 mol/L, indicating that the effect from osmotic pressure can promote biofilm formation. *B. longum* and *E. faecalis* could form biofilms well when the sodium chloride concentration was 0.05 mol/L; their biofilm masses were 1.65- and 1.42-times higher than those of control groups, respectively. The mixed-species biofilms showed a poor tolerance to high osmotic pressure. The biofilm mass slightly increased only when the concentration of sodium chloride was 0.05 mol/L. When the osmotic pressure was 0.15 mol/L and 0.2 mol/L, the mixed-species biofilm mass was significantly reduced to approximately three-fifths of that of the control group.

### 3.7. Antibiotic Tolerance of Mixed-Species Communities

The Table 1 shows the MBIC of different combinations. Among the mono-biofilms, *B. longum*, *B. ovatus,* and *L. gasseri* seemed to have similar sensitivity to CTX and AMP (MBIC ranged from 1 to 2 μg/mL). *E. faecalis* had stronger resistance to CTX than AMP, which was mainly due to the natural tolerance of it when faced with CTX. The MBIC of *E. faecalis* biofilms when faced with AMP and CTX was 1 μg/mL and 4 μg/mL, respectively. For two-strain biofilms, *B. longum* seemed to have a positive effect on improving antibiotic resistance in the communities. *B. ovatus* + *L. gasseri* had the worst antibiotic resistance; their MBIC values for CTX and AMP were 8 μg/mL and 32 μg/mL, respectively, which was mainly related to the low biomass and weak protection of the matrix. It is worth noting that under the protection of extracellular macromolecules, mixed-species biofilms showed greatly improved resistance to antibiotics. The MBIC of biofilms composed of these four strains was 256 μg/mL (AMP) and 128 μg/mL (CTX), revealing strong survivability.

### 3.8. Structural Characteristics of Biofilms

**Figure 7:**
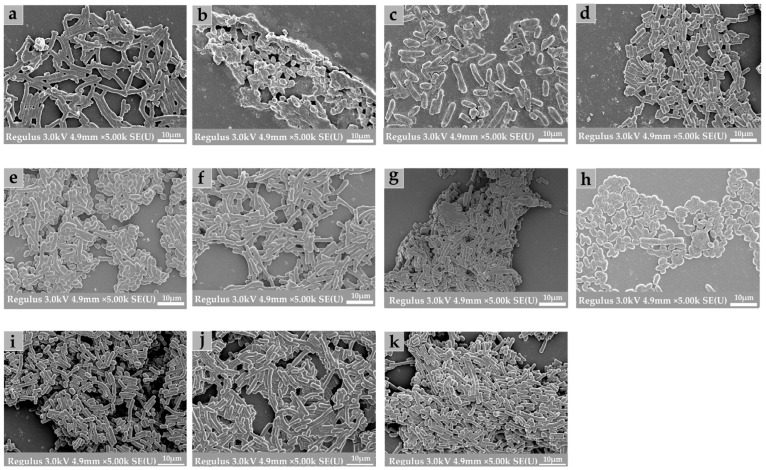
Field emission scanning electron microscopy (FESEM) images of mono- and mixed-species biofilms. All images were obtained at 5000× magnification. (**a**) *B. longum*; (**b**) *E. faecalis*; (**c**) *B. ovatus*; (**d**) *L. gasseri*; (**e**) *B. longum* + *E. faecalis*; (**f**) *B. longum* + *B. ovatus*; (**g**) *B. longum* + *L. gasseri*; (**h**) *E. faecalis* + *B. ovatus*; (**i**) *E. faecalis* + *L. gasseri*; (**j**) *B. ovatus* + *L. gasseri*; (**k**) *B. longum* + *E. faecalis* + *B. ovatus* + *L. gasseri*.

FESEM and CLSM were used to further explore the biological structure of mono- and mixed-species biofilms. As shown in Figure 7, the microstructure of biofilms composed of *B. longum*, *E. faecalis*, *B. ovatus*, and *L. gasseri* was investigated using scanning electron microscopy. Compared with the biofilms formed by mixed strains, mono-biofilms presented a sparse and dispersed structure, which was mainly subject to their weak biofilm-formation ability. According to the results of crystal violet Staining, minimum biofilm inhibitory concentration, and FESEM images, we can see that after co-cultivation of the different strains, both the biomass and the amount of extracellular matrix of mixed-species biofilms were significantly increased.

**Figure 8:**
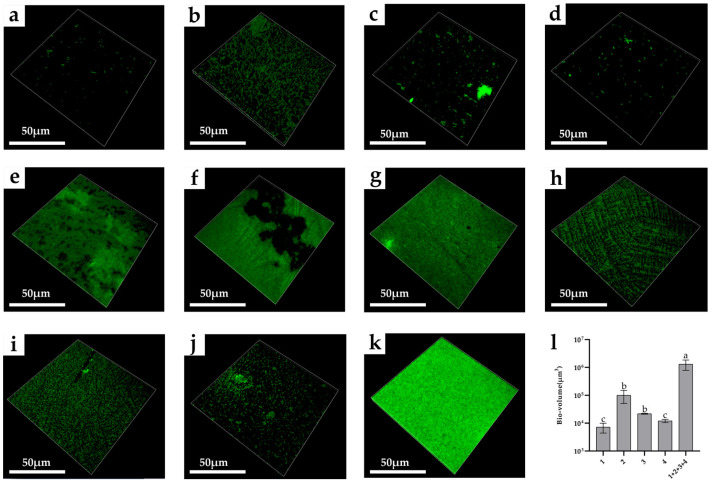
CLSM images of mono- and mixed-species biofilms. (**a**) *B. longum*; (**b**) *E. faecalis*; (**c**) *B. ovatus*; (**d**) *L. gasseri*; (**e**) *B. longum* + *E. faecalis*; (**f**) *B. longum* + *B. ovatus*; (**g**) *B. longum* + *L. gasseri*; (**h**) *E. faecalis* + *B. ovatus*; (**i**) *E. faecalis* + *L. gasseri*; (**j**) *B. ovatus* + *L. gasseri*; (**k**) *B. longum* + *E. faecalis* + *B. ovatus* + *L. gasseri*; (**l**) Bio-volume of mono- and mixed-species biofilms. 1—*B. longum*, 2—*E. faecalis*, 3—*B. ovatus* and 4—*L. gasseri*. Lowercase letters above the bars represent statistically significant differences between these groups (*p* < 0.05) after one-way ANOVA.

As shown in Figure 8, it can be seen through the confocal laser scanning microscope that the biofilm structure formed by the mixed strains was denser than that formed by the single strain. Among mono-biofilms, the bio-volume of *E. faecalis* was the highest (approximately 6.25 × 10^5^ μm^3^). After the co-cultivation of these species, the bio-volume of the four-strain biofilms increased. The bio-volume of the mixed-species biofilm was approximately 1.18 × 10^6^ μm^3^ (Figure 8l)

## 4. Discussion

Biofilms are dominant in various ecological niches; regardless of the ecological niche, biofilm communities are almost entirely composed of mixed strains and the complex interactions between these strains generally lead to mixed-species biofilms, exhibiting structural and functional characteristics that are different from those of mono-biofilms [30,31]. For example, biofilms formed by *Klebsiella pneumoniae*, *Pseudomonas aeruginosa*, *Proteus mirabilis*, and *Morganella morganii* have been identified in the infections of the urinary tract, and mixed microorganisms generally led to the aggravation of this disease [32]; on the other hand, the motility provided by the flagella of *Escherichia coli* can contribute to the formation of biofilms with *Vibrio cholerae*. Compared with mono-biofilms, their mixed biofilm had a denser structure. The increase in the growth of *Vibrio cholerae* may aggravate its infection in the human gut [33]. Therefore, it is of great significance to reveal the interaction of different species of bacteria in biofilms, which can be conducive to establishing new insight into gut mixed-species biofilms and treatment of infections caused by gut biofilms.

### 4.1. The Biofilms Formed by Gut Microbiota

The formation of a mixed-species biofilm is the result of ecological competition of different species and metabolic cooperation of related bacteria. For mixed-species biofilms, there are generally mutually beneficial relationships between different strains, which can support their own growth through metabolic activities. Therefore, the bacteria that have cross-feeding interactions may quickly aggregate during the formation of biofilms. For example, *A. tumefaciens*, *M. saperdae*, *M. saperdae,* and *O. anthropic*, which were capable of degrading metalworking fluids, exhibit strong support interactions in toxic environments [34], and Khoury found that polysaccharides produced by *C. albicans* were an important factor in mediating the formation of mixed-species biofilms with *S. mutans*. The polysaccharides were identified to promote the adhesion of *S. mutans* [35].

In the present study, when *B. longum*, *E. faecalis*, *B. ovatus*, and *L. gasseri* were co-cultured, the biofilm mass was the highest. The quantitative results show that each species is critical for biomass improvement. It is worth noting that *B. longum* was the key strain that contributed to the formation of mixed-species biofilms. Interestingly, when these four bacteria were cultured in isolation, *E. faecalis* had biofilm-formation ability, while *B. longum*, *B. ovatus* and *L. gasseri* could not form abundant biofilms

While exploring different bacterial metabolites, we found that metabolites also had a strong effect on the formation of mixed-species biofilms. Some vitamins, amino acids, and quorum-sensing signal molecules are produced by different strains fed with other viable cells. This can contribute to the formation of mixed-species biofilms. It should be noted that although the cell-free supernatant of *B. longum* had a promoting effect on biofilms of different combinations, the effect was not as good as that of viable cells. Therefore, the connection between *B. longum* and other bacteria was mainly the function of living cells.

### 4.2. Cell Number, Concentration of Extracellular Polysaccharide, Metabolic Activity, and MBIC of Mixed-Species Biofilms Increased

The biofilm formation ability of single species is very poor and these species mainly exist in planktonic cells. Therefore, it was of little significance to study the changes in the cell number of mono-biofilms. Given this circumstance, we explored the interactions between strains by quantifying cell numbers in the four-strain biofilm using qPCR. It can be seen from the result that *B. longum* and *L. gasseri* dominated in the biofilm, while *B. ovatus* and *E. faecalis* mainly existed as planktonic cells. All species in the mixed-species biofilms increased by at least three orders of magnitude, indicating that the formation of mixed-species biofilms benefited the growth of each strain.

The cell metabolic activity and concentration of extracellular polysaccharides in the mixed-species biofilm were further quantified using XTT reduction and anthrone-sulfuric acid methods. The ability of each strain to capture and metabolize nutrients was greatly improved in the mixed-species biofilms. Exopolysaccharides are key components of the extracellular matrix and are related to the adhesion and survival ability of biofilms [36,37]. By exploring the metabolic activity and concentration of exopolysaccharides in mono- and mixed-species biofilms, we can gain insights into the differences in the tolerance of different combinations to adverse environments.

In mixed-species biofilms, interactions between species play a key role in the success of the biofilm community, as some species depend on the metabolic activity of others for growth [38,39]. Various in vitro studies have shown that some strains that cannot form biofilms on their own can benefit from living with other species in communities [40]. An increase in polysaccharide content can improve the resistance of biofilms to antibiotics. A thick extracellular matrix can act as a physical barrier to prevent antibiotics, such as ampicillin and cefotaxime sodium, from penetrating the biofilm [41,42], which makes the structure of mixed-species biofilms stronger than that of mono-biofilms. For mono-biofilms, *E. faecalis* has a good tolerance to cefotaxime sodium, which is consistent with the previous reports [43]. Other strains seemed to be very sensitive to AMP and CTX, and their biofilms can be completely eradicated at very low antibiotic concentrations. For mixed-species biofilms, the presence of *B. longum* can significantly contribute to the increase in biofilm mass. Therefore, the mixed-species biofilms composed of *B. longum* were more resistant to AMP and CTX. In CTX experiment groups, the biofilms that were composed of E. faecalis had higher CTX resistance, which was mainly due to its good tolerance to CTX. However, E. faecalis did not dominate in mixed-species biofilm according to the results of qPCR. Therefore, the presence of E. faecalis failed to significantly increase the MBIC of mixed-biofilm composed of four bacteria.

### 4.3. Changes in Microstructure of Mixed-Species Biofilms

The FESEM and CLSM images can support our results that there is a synergistic interaction in mixed-species biofilms. Specifically, the CLSM images showed that the structure of mixed-species biofilm was stronger and denser than that of mono-biofilms. From the SEM images, it can be further found that the matrix of mixed-species biofilms mainly stuck together, while the structure of the mono-biofilm was relatively dispersed. The strengthened microstructure of mixed-species biofilms may be attributed to the improvement in metabolism and overexpression of biofilm-related genes [44]. This result may be the reason for the improvement in environmental resistance of mixed-species biofilms.

### 4.4. Microbial Biofilms and the Human Gut Microbiome

In the human intestine, biofilms usually form in the mucosa and lumen. There is evidence that biofilms play an important role in promoting the recovery of intestinal flora after antibiotic treatment, fecal microbiota transplantation, and in the face of an adverse environment [45]. For example, the biofilm formed by *Lactobacillus reuteri* and *Lactobacillus rhamnosus* has proved that the survival rate of cells in the biofilms was higher than their suspension cells after continuous gastrointestinal fluid exposure. Further, the number of bacterial cells in biofilms can recover quickly [46]. Except that, Aditya et al. explored the resistance and sensitivity of *Escherichia coli* to ciprofloxacin in vitro and found that a high concentration of ciprofloxacin will be needed if the biofilm of *E. coli* formed. All of this, mentioned above, was mainly due to the protection of extracellular polymeric substances (EPSs) in the biofilm matrix [47,48]. For probiotics and commensal bacteria, such as *B. longum*, *L. gasseri*, *E. faecalis* and *B. ovatus*, they have many competitive strategies to benefit from other competitors, such as limiting the growth space of other invaders through rapid growth [49]. This is a protective effect induced by biofilms to protect intestinal microbiota against pathogenic bacteria. Aside from that, many molecules are involved in these strategies, such as specific adhesion molecules, which are produced by the gut microbiota and can contribute to the improvement in adhesion ability [50]; extracellular polysaccharides can starve competitors and provide rich nutrition for settlers in biofilms [51]. We can expect more discoveries about the importance of biofilms in the stability and resilience of the gut microbiota.

## 5. Conclusions

We identified that *B. longum*, *L. gasseri*, *B. ovatus*, and *E. faecalis* had a strong synergistic effect on biofilm formation in a stable static culture system. The biological properties of mixed-species biofilms composed of these strains, such as concentration of extracellular polysaccharides, metabolic activity, and antibiotic tolerance, were stronger than those of mono-biofilms. The formation of mixed-species biofilms significantly improved the survival rate of each strain under different environments. In addition, *B. longum* is critically important in this community for promoting the improvement in these properties. Our result was a preliminary study of gut mixed-species biofilms and it is necessary to further explore the mechanisms underlying gut biofilm formation.

## Figures and Tables

**Table 1:** Minimum biofilm inhibitory concentration (MBIC) of mono- and mixed-species biofilms. AMP: ampicillin; CTX: cefotaxime sodium.

Biofilms	MBIC (μg/mL)	
	AMP	CTX
*B. longum*	2	1
*E. faecalis*	1	4
*B. ovatus*	1	1
*L. gasseri*	2	2
*B. longum* + *E. faecalis*	64	32
*B. longum* + *B. ovatus*	128	16
*B. longum* + *L. gasseri*	128	16
*E. faecalis* + *B. ovatus*	32	16
*E. faecalis* + *L. gasseri*	64	16
*B. ovatus* + *L. gasseri*	32	8
*B. longum* + *E. faecalis* + *B. ovatus*	64	64
*B. longum* + *E. faecalis* + *L. gasseri*	128	32
*B. longum* + *B. ovatus* + *L. gasseri*	128	32
*E. faecalis* + *B. ovatus* + *L. gasseri*	128	32
*B. longum* + *E. faecalis* + *B. ovatus* + *L. gasseri*	256	128

## Data Availability

Not applicable.

## Supplementary Materials

The following are available online at https://www.mdpi.com/article/10.3390/microorganisms10122332/s1, Table S1. Details of different strains used in this study. Table S2. Details of medium used in this study. Table S3. Primer sequences used in qPCR for species. Figure S1. Images of the crystal violet staining of different combinations. Figure S2. Standard curves of qPCR. References [52,53,54,55] are cited in the supplementary materials.
