# Supplementary material for: Characterization of Mixed-Species Biofilms Formed by Four Gut Microbiota
Source: Microorganisms. 2022 Nov 25;10(12):2332. doi: 10.3390/microorganisms10122332 (PMC9781930; doi:10.3390/microorganisms10122332)
Supplement: Supplementary file 1 [file microorganisms-10-02332-s001.zip › microorganisms-2016491-supplementary.pdf]

Characterization of Mixed-Species Biofilms Formed by Four Gut Microbiota

Tao Xu [1,2], Yue Xiao [1,2], Hongchao Wang [1,2], Jinlin Zhu [1,2], Yuankun Lee [3,4], Jianxin Zhao [1,2,5], Wenwei Lu [1,2,4,5,6,*] and Hao Zhang [1,2,5,6]

1 State Key Laboratory of Food Science and Technology, Jiangnan University, Wuxi 214122, China
2 School of Food Science and Technology, Jiangnan University, Wuxi 214122, China
3 Department of Microbiology & Immunology, Yong Loo Lin School of Medicine, National University of Singapore, Singapore 117545, Singapore
4 International Joint Research Laboratory for Pharmabiotics & Antibiotic Resistance, Jiangnan University, Wuxi 214122, China
5 (Yangzhou) Institute of Food Biotechnology, Jiangnan University, Yangzhou 225004, China
6 National Engineering Research Center for Functional Food, Jiangnan University, Wuxi 214122, China
* Correspondence: luwenwei@jiangnan.edu.cn; Tel.: +86-510-85912155

Table S1. Details of different strains used in this study.

Species	Number	label
Bifidobacterium longum subsp. Infantis	FJND16M4	A
Bifidobacterium longum subsp. Infantis	FBJCP1M11	B
Enterococcus faecalis	E1	C
Enterococcus faecalis	JNGMMB7	D
Bacteroides ovatus	FTJS5K9	E
Bacteroides ovatus	FNXYCHL6K1	F
Lactobacillus gasseri	FHNFQ11L7	G
Lactobacillus gasseri	FHNFQ14L5	H

Table S2. Details of medium (1L) used in this study.

Formulation	Content	
Casein peptone	10.00	g
tryptone	10.00	g
Yeast extract	2.50	g
Sodium acetate	5.00	g
Lactose × H_2O	5.00	g
Glucose	5.00	g
$MgSO_4$ × 7 H_20	45.00	mg
$CaCl_2$ × 2 H_20	90.00	mg
K_2HPO_4	0.45	g
KH_2PO_4	0.45	g
NaCl	0.90	g
Resazurin	1.00	mg
Distilled water	1000.00	mL
$NaHCO_3$	4.00	g
L-Cysteine-HCl	1.00	g
Hemin	10.00	mg

Cellobiose	2.00	g
$(NH_4)_2SO_4$	0.90	g
Biotin	0.01	mg
Vitamin B_{12}	0.10	mg
p-Aminobenzoic acid	5.00	mg
Folic acid	2.00	mg
Pyridoxine-HCl	10.00	mg
Vitamin B_1	0.05	mg
Riboflavin	0.05	mg
D-Ca-pantothenate	5.00	mg
Lipoic acid	5.00	mg
Nicotinic acid	2.00	mg
Acetic acid	1.50	mL
Propionic acid	0.70	mL
Iso-Butyric acid	90.00	μL
n-Valeric acid	100	μL
Iso-Valeric acid	100.00	μL
Clarified rumen fluid	10	mL
PH	6.2 – 6.4	

Table S3. Primer sequences used in qPCR for species.

Species	Primer name	Sequence (5′-3′)	Amplicon size (bp)	Reference
Bifidobacterium longum subsp. Infantis	*B. longum*_F<br>*B. longum*_R	ATCCGTCCGACCCAGACC<br>CTCGACATCCTCACGGCC	123	[1]
Enterococcus faecalis	*E. faecalis*_F<br>*E. faecalis*_R	CGCTTCTTTCCTCCCGAGT<br>GCCATGCGGCATAAACTG	143	[2]
Bacteroides ovatus	*B. ovatus*_F<br>*B. ovatus*_R	AAGACAACGATGGATAGGGGTT<br>TTCACGCTACTTGGCTGGT	136	[3]
Lactobacillus gasseri	*L. gasseri*_F<br>*L. gasseri*_R	AGCGAGCTTGCCTAGATGAATTTG<br>TCTTTTAAACTCTAGACATGCGTC	171	[4]

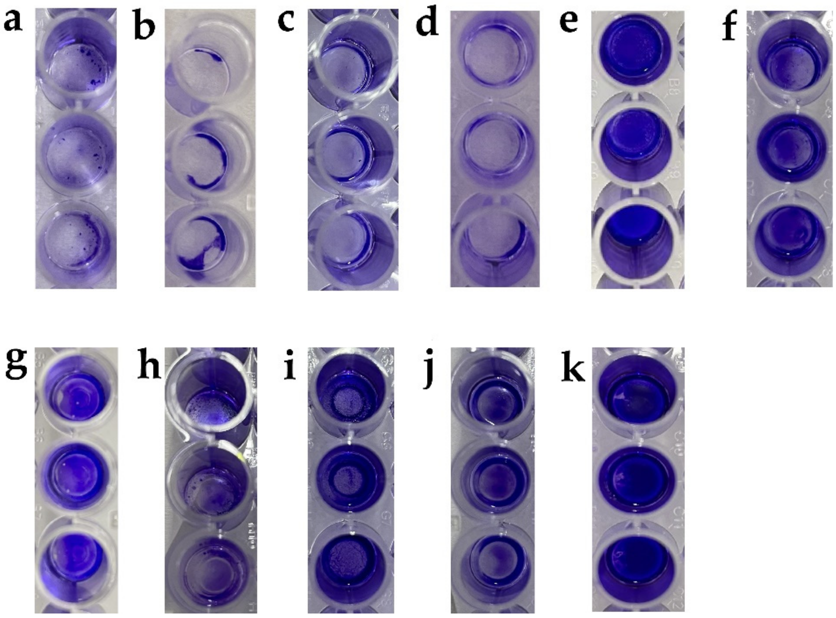

Figure S1. Images of the crystal violet staining of different combinations. (a) *B. longum*; (b) *E. faecalis*; (c) *B. ovatus*; (d) *L. gasseri*; (e) *B. longum* + *E. faecalis*; (f) *B. longum* + *B. ovatus*; (g) *B. longum* + *L. gasseri*; (h) *E. faecalis* + *B. ovatus*; (i) *E. faecalis* + *L. gasseri*; (j) *B. ovatus* + *L. gasseri*; (k) *B. longum* + *E. faecalis* + *B. ovatus* + *L. gasseri*.

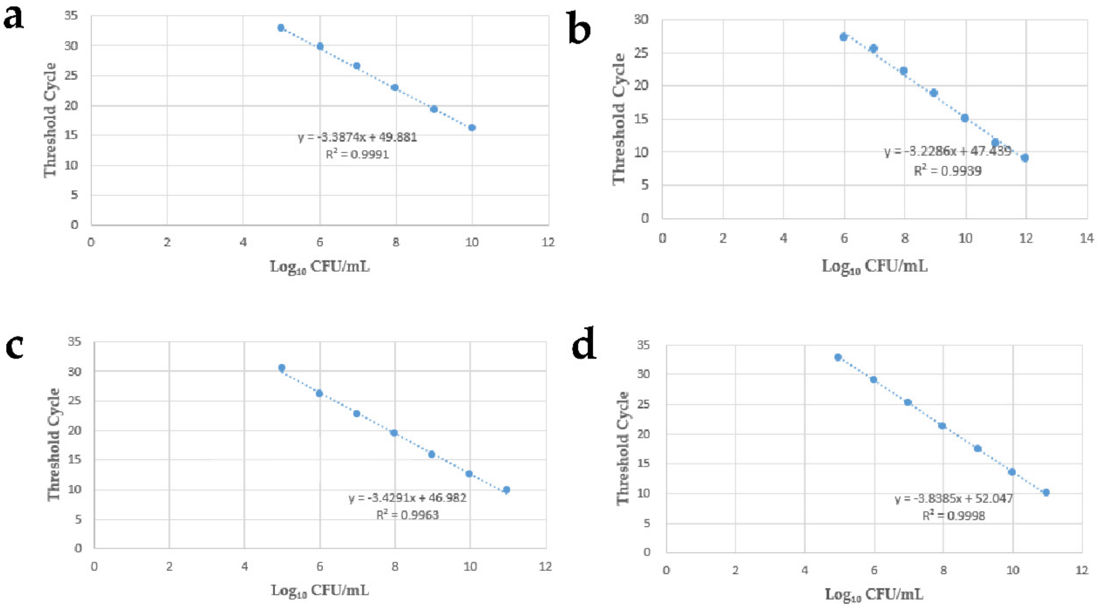

Figure S2. Standard curves of qPCR. (a) *B. longum*. (b) *E. faecalis*. (c) *B. ovatus*. (d) *L. gasseri*.

Reference

[1] Sheu, S.-J.; Hwang, W.-Z.; Chiang, Y.-C.; Lin, W.-H.; Chen, H.-C.; Tsen, H.-Y. Use of Tuf Gene-Based Primers for the PCR Detection of Probiotic Bifidobacterium Species and Enumeration of Bifidobacteria in Fermented Milk by Cultural and Quantitative Real-Time PCR Methods. *J. Food Sci.* **2010**, *75*, M521–M527, https://doi.org/10.1111/j.1750-3841.2010.01816.x.

[2] Heidar, S.A.-H.A.; Saber, S.E.; Eissa, S.A.-L.A.-A.; El-Ashry, S.H. Antibacterial potential of nano-particulate intracanal med-ications on a mature E. faecalis biofilm in an ex-vivo model. *Giornale Italiano di Endodonzia*. **2020**, *34*, 100-109. https://doi.org/10.32067/GIE.2020.34.02.04.
[3] Gu, X.; Yu, T.; Guo, T.; Kong, J. A qPCR-based method for rapid quantification of six intestinal homeostasis-relevant bacterial genera in feces. *Futur. Microbiol*. **2021**, *16*, 895–906, https://doi.org/10.2217/fmb-2020-0269.
[4] Kim, J.H.; Yoo, S.M.; Sohn, Y.H.; Jin, C.H.; Yang, Y.S.; Hwang, I.T.; Oh, K.Y. Predominant Lactobacillus species types of vaginal microbiota in pregnant Korean women: quantification of the five Lactobacillus species and two anaerobes. *J. Matern. Neonatal Med*. **2016**, *30*, 2329–2333, https://doi.org/10.1080/14767058.2016.1247799.
